# Cirugía estética y “terapéutica psicológica” en Argentina durante la primera mitad del siglo XX

**DOI:** 10.1590/S0104-59702025000100063

**Published:** 2026-02-09

**Authors:** Joaquín Molina

**Affiliations:** i Profesor, Instituto de Altos Estudios Sociales, Universidad Nacional de San Martín. San Martín – Buenos Aires – Argentina. joaquinmolina079@gmail.com

**Keywords:** Cirugía estética, Psicología, Complejo de inferioridad, Argentina, Siglo XX, Aesthetic surgery, Psychology, Inferiority complex, Argentina, Twentieth century

## Abstract

El artículo se propone analizar la construcción de la cirugía estética como “terapéutica psicológica” entre los cirujanos plásticos argentinos en la primera mitad del siglo XX. Para ello, se analizan publicaciones médicas expertas y de divulgación producidas por estos especialistas. El trabajo comienza planteando una controversia jurídica en torno al valor terapéutico de la cirugía estética. Partiendo de esta controversia, se analizan las justificaciones psicológicas esgrimidas en la literatura médica y algunas narrativas de casos que dan cuenta del carácter “preventivo” de estas prácticas. Al cierre, se abordan las iniciativas de los cirujanos plásticos argentinos destinadas a asegurar el acceso económico a la cirugía estética.

## Abstract (en)

This article aims to analyze the construction of aesthetic surgery as a “psychological therapy” among Argentine plastic surgeons in the first half of the twentieth century. To this end, it analyzes expert and popular medical publications produced by these specialists. The article begins by raising a legal controversy surrounding the therapeutic value of aesthetic surgery. Based on this controversy, it analyzes the psychological justifications put forward in the medical literature and some case narratives that demonstrate the “preventive” nature of these practices. It concludes with a discussion of the initiatives undertaken by Argentine plastic surgeons to ensure affordable access to aesthetic surgery.

## Cirugía estética: ¿práctica banal o terapéutica psíquica?

El 27 de febrero de 1928, el cirujano francés Charles Dujarier llevó adelante una intervención quirúrgica destinada a reducir el grosor de las piernas de Suzanne Geoffre, propietaria de una casa de moda situada en París. En las semanas que siguieron a la cirugía, la gangrena avanzó sobre el miembro derecho de la paciente, lo que motivó a que Dujarier tuviera que amputarle el tercio inferior de la pierna. Tiempo después, la mujer presentó una demanda civil, reclamando quinientos mil francos de indemnización en concepto de daños y perjuicios. El 20 de febrero de 1929, el Tribunal Civil del Sena condenó al cirujano a pagar doscientos mil francos. El magistrado argumentó su fallo sosteniendo que “el hecho de haber emprendido una operación que comportaba un riesgo grave, sobre un miembro sano… sin que esta intervención fuera impuesta por una necesidad terapéutica… constituye por sí mismo una falta de tal naturaleza que entraña la responsabilidad del Cirujano” (Claoué, Bernard, 1936, p.7). La sentencia generó un amplio revuelo en la comunidad médica y extra-médica, alcanzando las páginas de la prensa francesa (Comiskey, 2004).

El caso Dujarier también suscitó debates en el campo del derecho argentino. El 29 de agosto de 1929, el médico legista Nerio Rojas (1929, p.14) publicó un artículo en el que señala que es injusto considerar “estas intervenciones como lujos más o menos innecesarios… porque un defecto físico marcado tiene inconvenientes morales, sentimentales y hasta económicos” que confieren un carácter terapéutico a estas prácticas. Una postura diametralmente opuesta es la sostenida por Juan Silva Riestra. A criterio de este especialista en derecho penal, cuando el médico interviene “no con el propósito de curar una enfermedad o de corregir una deformidad repulsiva sino con un fin de ‘embellecimiento’ del sujeto… es responsable de sus consecuencias” (Silva Riestra, 1938, p.5). Casi veinte años después, el caso Dujarier continuaría alimentando la polémica entre los profesionales del derecho. Así lo deja entrever el médico legista José Belbey. Refiriendo al caso en cuestión, argumenta que lejos de ser un “arte menor” cuyo “peligro” supera con creces el “beneficio obtenido”, la cirugía estética es “curativa” al corregir “defectos físicos” que originan “enfermedades mentales” asentadas en un “complejo de inferioridad” (Belbey, 1957, p.156).

El caso Dujarier, y sus derivas en el campo del derecho argentino, permite circunscribir analítica e históricamente la problemática que será abordada en este trabajo. Analíticamente, el caso exhibe el carácter controversial de las prácticas de cirugía estética. Históricamente, el fallo de la justicia parisina parecería constituir un punto de inflexión a partir del cual las producciones escritas tendentes a difundir y legitimar las prácticas de cirugía estética se multiplican y adquieren mayor visibilidad. Así, refiriéndose al contexto francés, Le Hénaff (2017, p.16) señala que “los discursos orientados a defender las ventajas terapéuticas de la cirugía estética… asumieron mayor visibilidad cuando fueron empleados en la defensa del cirujano”. En Argentina, este fenómeno parecería replicarse, en la medida en que la mayor parte de las publicaciones médicas recogidas y analizadas en este artículo se sitúan entre fines de la década de 1920 y comienzos de la de 1950.

En este marco, me propongo analizar las publicaciones médicas expertas y de divulgación producidas por cirujanos plásticos argentinos de la primera mitad del siglo XX a los efectos de identificar el modo en que aspiraron a construir la estética corporal como fuente de problemas psicológicos y a la cirugía estética como una terapéutica eficaz capaz de responder a dichos problemas. En la primera parte del artículo, realizo un repaso por los principales hitos históricos que confluyeron en la conformación e institucionalización de la cirugía plástica argentina durante la primera mitad del siglo XX. En la segunda parte, ingreso de lleno en el análisis de la literatura médica. En primer lugar, veremos que las justificaciones psicológicas en torno a estas prácticas remiten a inicio del siglo XX, pero que sería recién entre la década de 1930 y principios de la de 1940 que en Argentina se articularía un saber psicológico en torno a estos padecimientos a partir de la difusión de la noción de “complejo de inferioridad”. En segundo lugar, me propongo abordar la principal evidencia articulada por los cirujanos plásticos argentinos para conferir un carácter terapéutico a sus prácticas, colocando el foco de análisis en algunas narrativas de casos sobre cirugías estéticas de las orejas en niños y adolescentes. Según argumento, estas narrativas permiten vislumbrar el carácter “preventivo” y “reformador” que los cirujanos pretendían imprimir a sus prácticas, así como el carácter biopolítico que asumirían estas intervenciones al permitir la integración virtuosa de los pacientes operados al orden social. Al cierre, analizo la postura de los cirujanos estéticos argentinos en torno a la cirugía estética como un “derecho”, así como las distintas iniciativas tendentes a democratizar el acceso a esta “terapéutica psíquica”.

## Historia de la cirugía plástica en Argentina

Las primeras publicaciones argentinas en materia de cirugía plástica datan de fines del siglo XIX y principios del XX. Con algunas excepciones, una buena proporción de ellas versa sobre reconstrucción de nariz. El advenimiento de la Primera Guerra Mundial marcaría un punto de inflexión en la historia internacional y nacional de la cirugía plástica. La literatura académica coincide en destacar que, junto al elevado número de contendientes lesionados en el tronco y las extremidades, el conflicto bélico dejó como saldo una elevada proporción de traumatizados de la cabeza y el rostro. Fue en este escenario en el que se presentó la acuciante necesidad de organizar servicios especializados en este tipo de lesiones, cuya afluencia en volumen y gravedad impulsó el desarrollo de la cirugía plástica maxilofacial. Los centros que surgieron al calor del conflicto fueron puestos en manos de cirujanos con escasa formación en la materia que mediante prueba y error dieron lugar a una serie de innovaciones técnicas y organizacionales en materia de reconstrucción del rostro (Gehrhardt, 2015).

Dichos avances médicos contribuirían al desarrollo de la cirugía plástica en Argentina, gracias a la circulación internacional de personas y conocimientos médicos. Uno de los hechos más destacables fue la gira latinoamericana realizada por el cirujano francés León Dufourmentel en 1932. Enviado por el Ministerio de Salud Pública de Francia, este especialista en cirugía maxilofacial redactó un informe en el que señala que “América del Sur, tal vez porque no ha sufrido la prueba de la gran guerra, no ha conocido el esfuerzo de la cirugía reparadora como Europa o los Estados Unidos de América”. No obstante, el francés destaca que los “cirujanos argentinos” parecen tener “suficiente empuje y aptitudes para ponerse rápidamente al nivel y mismo a la cabeza del movimiento” de expansión de la cirugía plástica en Latinoamérica (Dufourmentel, 1933, p.357).

En el período de entreguerras también comenzaron a surgir los primeros centros especializados en cirugía plástica y a perfilarse los cirujanos en torno a los cuales gravitaría la institucionalización de la disciplina en Argentina. Desde ya, que se trató de un proceso paulatino en el que médicos que ejercían en servicios de cirugía general o de atención especializada en hospitales públicos (principalmente, traumatología y otorrinolaringología) se aventuraban en el terreno de un quehacer quirúrgico poco conocido. Prácticamente la totalidad de este movimiento se centraliza en Buenos Aires y, en menor medida, en la ciudad de Rosario (provincia de Santa Fe). Asimismo, aunque el grueso de la actividad en los servicios hospitalarios se focalizaba en la cirugía reconstructiva, durante la década de 1920 también comenzaron a practicarse cirugías estéticas. Con elevada frecuencia, estas cirugías se realizaban sobre el rostro, adquiriendo predominio las intervenciones sobre la nariz. Finalmente, cabe destacar que, junto al ejercicio de estas prácticas en el sector público, también se registran algunas referencias a la actividad en sanatorios y clínicas particulares.

Entre fines de la década de 1930 y principios de la de 1940 se producirían dos eventos de relevancia para la historia de la cirugía plástica internacional y latinoamericana. El advenimiento de la Segunda Guerra Mundial reactualizaría los desafíos a la todavía naciente disciplina. Según Mayhew (2004), el alto costo que implicó la guerra de trincheras llevó al gobierno inglés a centrar buena parte de sus esfuerzos en una estrategia ofensiva que confiriera un mayor peso a las fuerzas aéreas. El reverso de este cambio táctico fue la emergencia de un conjunto de heridos que por la similitud de las causas y de las patologías que presentaban adquirieron denominación médica propia: *airman’s burn* [quemadura de aviador]. La atención de estos casos de nuevo tipo impulsaría un cambio en la terapéutica de las quemaduras y estructuraría una organización que propiciara la puesta en práctica de dicho cambio (Kiehn, 1995).

Durante el período mencionado, se producirían numerosos eventos significativos relacionados a la circulación internacional de personas y de conocimientos médicos. Por su relevancia para la historia de la cirugía plástica latinoamericana, cabe destacar el arribo a la Argentina de Herbert O. Bames, cirujano plástico de Los Ángeles (California). En su paso por el país, operó en varios servicios hospitalarios de Buenos Aires y Rosario. Según rememora Ernesto Malbec (1943, p.58), en el discurso de apertura al segundo Congreso Latinoamericano de Cirugía Plástica (realizado en 1942 y publicado en 1943): “[Bames] insistió reiteradamente sobre la necesidad de constituir una sociedad… que agrupara en su seno a todos los especialistas del continente”. A instancias del norteamericano, los distintos cultores de la disciplina a nivel latinoamericano iniciaron una serie de intercambios epistolares para llevar adelante la idea. En julio de 1940, bajo el impulso de los cirujanos brasileros Antonio Prudente y Rebelo Neto, se llevó a cabo en San Pablo una reunión de los principales representantes latinoamericanos de la cirugía plástica dando constitución formal a la entidad que los nuclearía.

Doce años deberían transcurrir entre la fundación de la entidad latinoamericana y la conformación de la Sociedad Argentina de Cirugía Plástica (SACP). El germen de la entidad surge en 1949, en el marco de una serie de ateneos hospitalarios realizados en las principales secciones o servicios de Buenos Aires en los que se practicaba cirugía plástica. En 1951, al cierre de un ateneo que tuvo lugar en el Hospital Argerich, se realizó una reunión en la que designó una Comisión Provisoria que tendría por objetivo redactar un anteproyecto para la organización de la Sociedad de Cirugía Plástica. Este último se discutió y se votó punto por punto en tres Asambleas Extraordinarias que tuvieron como sede el local de la Asociación Médica Argentina. En la tercera, que tuvo lugar el 24 de marzo de 1952, se firmó el “Acta de fundación” y es considerada como el momento en que vio la luz oficialmente la SACP (Patané, 1986). En muy poco tiempo, dicha entidad experimentaría un incremento vertiginoso: en el período 1953-1961 la SACP pasa de tener 68 miembros a totalizar 159, lo que muestra que en solo ocho años se produjo un incremento del 133% de la masa societaria.

Este apartado permitió identificar los principales hitos vinculados a la emergencia y consolidación institucional de la cirugía plástica argentina durante la primera mitad del siglo XX. A continuación, procuraremos identificar y describir el modo en que los promotores de la naciente disciplina buscaron delinear una esfera de problemas psicológicos que confirieran legitimidad a sus prácticas y aspiraron a brindar evidencia en torno a la eficacia de las intervenciones estéticas para resolverlos.

## La cirugía estética y el complejo de inferioridad

En 1940, el médico clínico cordobés Jorge Orgaz publicó en la revista *El Día Médico* un breve e incisivo artículo titulado “Cirugía estética”, a la que define como “refugio y engaño de nuestro narcicismo confesable y tolerable. Ortopedia más para el parecer que para el ser” (Orgaz, 29 ene. 1940, p.83). Dicho artículo parece haber tocado ciertas fibras sensibles de los cirujanos plásticos, al punto de ser retomado por Lelio Zeno (1943, p.31-31) en el discurso inaugural del segundo Congreso Latinoamericano de Cirugía Plástica: “[Existe] en el mismo ambiente médico, una visión parcial y a veces equivocada de su obra. Se la supone, en efecto, antes que una ciencia, un arte o una técnica narcisista al servicio ‘más del parecer que del ser’, como ha dicho Jorge Orgaz”. Zeno contesta a esta crítica, destacando que las “desarmonías estéticas” tienen una “repercusión patógena” y que su corrección impacta en la “personalidad psicofísica” de los pacientes.

En línea con ello, durante la primera mitad del siglo XX, los cirujanos plásticos argentinos procuraron responder a este tipo de críticas dando cuenta de los problemas psicológicos asociados a los “defectos” corporales. Desde ya, que la articulación de fundamentos psicológicos en torno a prácticas aparentemente banales no fue un proceso que remite únicamente a los especialistas argentinos. En línea con ello, refiriendo al desenvolvimiento de la cirugía estética en Norteamérica, Gilman (1998) muestra las distintas teorías psicológicas articuladas en torno a la eficacia terapéutica de estas prácticas durante buena parte del siglo XX. Según argumenta, dichas teorías adoptan un enfoque “somato-psíquico”, en el que la corrección de las “anomalías físicas” devienen en una cura para los pacientes psicológicamente “infelices”. La misma idea es retomada por Haiken (1997) en su libro sobre la historia de la cirugía plástica en Estados Unidos, en el que destaca la centralidad que asumió la noción de “complejo de inferioridad” para articular una racionalidad terapéutica en torno a la cirugía estética. Para el contexto europeo, Guirimand (2005) reconstruye la historia de la cirugía estética en Francia durante el período de entreguerras, destacando que para los pioneros de la disciplina la “normalización” física de los pacientes podía jugar un rol importante en la profilaxis del suicidio y la cura de los neuróticos. A nivel latinoamericano, varias investigaciones brasileñas sobre el desenvolvimiento contemporáneo de la cirugía estética ponen de relieve el lugar del “bienestar” y la “calidad de vida” en la construcción de una concepción ampliada de la salud. Según argumentan, dicha concepción contribuye a cuestionar el límite entre estética y reparación, justificando las modificaciones cosméticas en términos de un derecho a la belleza (Antonio, 2008; Emonds, 2010; Edmonds, Sanabria, 2016; Schimitt, Rohden, 2020).

En la literatura médica argentina sobre cirugía plástica, las referencias a los problemas psicológicos asociados a la apariencia corporal y a la eficacia de la cirugía estética para resolverlos tienen larga data. Así, en fecha temprana como 1910, Pedro Tesone (1910, p.28) destaca la necesidad de efectuar reconstrucciones nasales a partir de inyecciones de parafina fundándose en el hecho de que este tipo de defectos puede “impedir la realización de proyectos”, produciendo “alteraciones psíquicas y por ende ideas de suicidio”. Por su parte, hacia fines de la década de 1920, el cirujano argentino Alejandro Forero (1929, p.1462-1463) señala que el excesivo desarrollo de la nariz conduce a sus portadores a una “depresión psíquica” cuyas manifestaciones más evidentes son las “tentativas de suicidio”, los “duelos provocados por las burlas que originan” y la inclinación por “la vida de claustro”. En buena parte del corpus empírico correspondiente a la década de 1920 y la primera mitad de la de 1930 se observa un uso impreciso de las nociones psicológicas y una alternancia entre estas nociones y referencias a la “moral” o al “alma” de los “defectuosos”. Así, por ejemplo, es habitual encontrar en un mismo texto expresiones que describen alternativamente el estado de los pacientes como “problemas de índole psíquica”, “sufrimiento moral” o “estados afligentes del alma”.

Estos primeros intentos orientados a medicalizar (Conrad, Schneider, 1992) la experiencia de las personas portadoras de defectos estéticos y a escenificar el carácter terapéutico de la cirugía estética adquieren mayor elaboración a partir del trabajo de colaboración psicológico-quirúrgico iniciado por Lelio Zeno (cirujano plástico) y Emilio Pizarro Crespo (médico psicólogo) en el Hospital Centenario de Rosario en la década de 1930. Lelio Zeno (1890-1968) fue un médico cirujano de orientación comunista que ejerció buena parte de su carrera en la ciudad de Rosario. Egresado como médico en 1915 en la Universidad de Buenos Aires, durante la década de 1920 realizó varios viajes de formación a Estados Unidos y Europa, experiencias que terminarían por decantarlo hacia la ortopedia y la traumatología. Habiendo acumulado cierto nivel de reconocimiento en la materia, en 1931 fue invitado por el director del Instituto Sklifosovsky (Moscú, URSS) para asumir la tarea de reorganizar el servicio de traumatología de dicho centro de emergencias médicas. Entre 1935 y 1936, Zeno realizaría un segundo viaje a la URSS, para dirigir el Centro de Traumatología en el Hospital Basmania. Al regreso de su segunda estancia en la URSS, el cirujano comenzó a mostrar un marcado interés por la cirugía plástica (Prieto, 2021). El resultado de su febril labor en la novel disciplina quedaría plasmado con la publicación del libro *Cirugía plástica* en 1943.

Emilio Pizarro Crespo (1904-1944) fue un médico de orientación comunista que egresó en la Universidad Nacional de Córdoba y practicó la psicoterapia en Rosario. Fue miembro asociado de la Sociedad Psicoanalítica de París y secretario de redacción de la revista *Psicoterapia* fundada en 1936 por el psicólogo de izquierda Gregorio Bermann (Vezzetti, 1996). Su obra se sitúa en un contexto de crisis del positivismo y del modelo somático. Al igual que otros médicos de izquierda, intentó “utilizar el psicoanálisis como una herramienta de modernización de la psiquiatría y como una metodología de crítica social” (Plotkin, 2001, p.28). Además de sus aportes vinculados a la difusión del psicoanálisis y de la medicina psicosomática, también se destacan algunos trabajos en los que intentó combinar a Freud y Marx. Simpatizante del comunismo, en 1935 visitó la URSS y en 1937 se trasladó a España para incorporarse al bando republicano. Poco antes de morir, en 1944, realizó un giro abrupto en su ideología política publicando un panfleto nacionalista de derecha que llevó por título “Afirmación gaucha” (Falcone, 2007).

A mediados de la década de 1930, Pizarro Crespo público su primer libro sobre medicina psicosomática dedicado a las alergias, en el que destaca la ineficiencia de la medicina tradicional para identificar y tratar los factores psicógenos que están en el origen de estos problemas orgánicos. Durante este período, inicia su colaboración con el cirujano plástico Lelio Zeno, que daría sus frutos en varios artículos publicados en prestigiosas revistas médicas argentinas y que serían retomados en los libros *Cirugía plástica* (Zeno, Pizarro Crespo, 1943) y *Clínica psicosomática* (Zeno, Pizarro Crespo, 1945). En esta última obra, los autores exhiben una pretensión didáctica al procurar transmitir métodos de observación y análisis clínico del enfermo que rompa con la “concepción materialista” de la “medicina académica”. Zeno y Pizarro Crespo dedican los tres últimos capítulos del libro al análisis e interpretación psicosomática de las historias clínicas de pacientes de cirugía plástica, con el objetivo de identificar las repercusiones que los “dismorfismos” tienen sobre la personalidad psico-física de los pacientes. Para ello, se apoyan en los saberes psicológicos en boga durante este período, cuya articulación está marcada por un fuerte eclecticismo (Plotkin, 1996).

De este conjunto de referencias, me gustaría destacar una noción que tuvo particular trascendencia en el campo de la cirugía plástica: el “complejo de inferioridad”, noción acuñada por Alfred Adler (1870-1937). Este último fue un médico psiquiatra que en 1902 estableció vínculos de colaboración con Freud, constituyéndose en uno de los cuatro fundadores de la Sociedad Psicoanalítica de Viena. En 1911, debido a las fuertes disidencias que mantenía con el padre del psicoanálisis, renunció a la organización y fundó la Sociedad de Psicología Individual (Bret, 1997). La teoría del sentimiento y el complejo de inferioridad, que comenzó a prefigurarse en 1907 y adquirió forma definitiva entre 1909 y 1911, hace referencia al sentimiento de inadecuación e inseguridad derivadas de deficiencias físicas o psicológicas que pueden adquirir expresión en el retraimiento o en la sobrecompensación agresiva de las personas afectadas (Ganz, 2013).

El “complejo de inferioridad” tuvo una amplia acogida en la sociedad norteamericana desde fines de la década de 1920, al punto de devenir no sólo en una expresión recurrentemente utilizado en la literatura médica sobre cirugía plástica, sino también en un ícono de la cultura popular en el contexto de la Gran Depresión. Esta noción asumiría tal ubicuidad que aparecería citada en textos médicos, en la prensa, en registros clínicos e inclusive en las cartas de algunos pacientes. Según Haiken (1997), la popularidad de esta teoría obedeció al estilo coloquial de Adler y a la orientación fuertemente pragmática de la psicología individual. La teoría del complejo de inferioridad fue adoptada por los cirujanos plásticos norteamericanos entre las décadas de 1920 y 1930, deviniendo en una categoría que permitió dar cuenta de los problemas psicosociales que planteaban los defectos estéticos y la capacidad de la cirugía estética de curar estas dolencias mediante la normalización física de los pacientes (Gilman, 1998).

Igual trascendencia asumiría esta noción a nivel latinoamericano, en cuya difusión asumirían un papel predominante Zeno y Pizarro Crespo. Relevante en esta empresa de divulgación fue su participación en el segundo Congreso Latinoamericano de Cirugía Plástica (1942). El primero, en su calidad de presidente del Congreso, y el segundo como miembro titular de la entidad y expositor de un importante trabajo titulado “Sentimiento y complejo de inferioridad”. En esta alocución, Pizarro Crespo se propone precisar dichas nociones con el fin de brindar al auditorio de cirujanos plásticos los rudimentos conceptuales y prácticos para la construcción psicológica de la indicación en cirugía plástica. Según el autor, dicho equipamiento conceptual debería rectificar la mirada puramente “exterior o morfológica de la cirugía correctora”, para dar relevancia a las “repercusiones psicofísicas” de los problemas estéticos (Pizarro Crespo, 1943, p.317).

Para no agotar al lector con la reproducción exhaustiva de las referencias a esta expresión en los congresos de la Sociedad Latinoamericana de Cirugía Plástica, me gustaría centrarme en un pasaje que pone a las claras el nivel de aceptación de la teoría adleriana entre los cirujanos plásticos latinoamericanos a fines de la década de 1940: “No voy a insistir, porque es del conocimiento de quienes me escuchan, sobre el alcance de los trabajos de Adler en especial aquellos que se refieren al concepto de inferioridad fundado en defectos orgánicos … [En] todos estos casos la intervención del cirujano plástico tendrá una beneficiosa influencia sobre el carácter del individuo” (Más de Ayala, 1949, p.360). Quien pronunció estas palabras es el psiquiatra uruguayo Isidro Más de Ayala en el cuarto Congreso de la Sociedad Latinoamericana de Cirugía Plástica (1949). En ese trabajo, el autor apela a una extensa cita directa extraída del libro *Clínica psicosomática* (Zeno, Pizarro Crespo, 1945) para sustentar su posición.

En Argentina, pude hallar una referencia temprana a esta noción en un artículo de Rómulo Gil (1919) sobre prótesis oculares publicado en *La Semana Médica*. Sin embargo, recién a fines de la década de 1930 y principios de la de 1940, el “complejo de inferioridad” parece instalarse definitivamente en el vocabulario médico de los cirujanos plásticos argentinos. Sintomático de ello, es la utilización recurrente de esta expresión en varias de las publicaciones médicas del período. Por citar algunos ejemplos: en 1939, Ernesto Malbec (1939b, p.716) señala que “hay defectuosos físicos, que han hecho con el andar del tiempo un complejo intelectual de inferioridad”; en 1943, el cirujano Fernando Schreiber (1943, p.296) sostiene que las mujeres con senos hipertrofiados “desarrollan complejos de inferioridad”; en 1946, el cirujano argentino Ramón Palacio Posse (1946, p.145) sostiene que el estigma que recae sobre los defectuosos de las orejas “es un motivo más que suficiente para producir en sus portadores un verdadero complejo de inferioridad”.

Según hemos visto hasta aquí, una de las críticas frecuentemente formuladas a la cirugía estética es que se trata de prácticas banales que responden a una demanda narcisista de los pacientes. Pioneros y divulgadores de una especialidad médica disputada, los cirujanos procuraron delinear una esfera de problemas psicológicos asociados a la estética corporal y escenificar sus prácticas como intervenciones que al modificar la apariencia inciden sobre la inferioridad de los pacientes (Gilman, 1998). En el próximo apartado, me propongo examinar el modo en que estos profesionales despliegan evidencia médica destinada a fundamentar el carácter curativo de sus prácticas, colocando el foco de análisis en las narrativas de casos presentes en la literatura médica de la primera mitad del siglo XX.

## Cirugía estética de las orejas como prevención del comportamiento “desviado”

En una investigación apoyada en el análisis de emisiones televisivas sobre cirugía plástica en Francia durante el período 1952-2008, las investigadoras Barbot y Cailbault (2010) procuran identificar los cambios históricos vinculados a la legitimación de la cirugía estética y el trabajo de figuración de las personas que recurren a ella. Según afirman, en los años 1950 primaba un discurso en el que la cirugía estética contribuía a “normalizar” a personas cuyos defectos corporales “objetivos” actuaban como un impedimento para sus posibilidades de inserción y éxito social. En este marco, la corrección quirúrgica permitiría sustraer a las personas de su condición de víctimas y modificar sus comportamientos gracias a la eliminación de los problemas psicológicos producidos por una sociedad estigmatizante.

El análisis de la literatura médica argentina de la primera mitad del siglo XX deja entrever una coincidencia punto por punto con el planteo de las investigadoras francesas. Dicha correspondencia se torna palpable en las narrativas de casos que, apelando al contraste entre el “antes” y el “después”, pretenden mostrar el problema psicológico que aquejaba a los pacientes y los cambios positivos experimentados en el posoperatorio. Usualmente, estas narrativas reconstruyen la trayectoria del paciente hasta el momento de arribar a la consulta. Cada una de las etapas de esta trayectoria está marcada por la hostilidad de los “normales” que se manifiesta en distintos espacios públicos a través de gestos y designaciones denigrantes. La experiencia de humillación pública de los “defectuosos” constituye la contracara exacta de estas reacciones sociales y usualmente tiene como correlato dos tipos de conductas: el repliegue en la esfera doméstica y un resentimiento creciente que puede derivar en explosiones de violencia. Ante este panorama, la cirugía estética emerge como una práctica que, mediante la “normalización” física, previene la articulación de comportamientos “desviados” y contribuye a la integración social de los operados.

Para darle consistencia empírica a lo expuesto hasta aquí, considero conveniente analizar una serie de narrativas que refieren a cirugías estéticas de orejas en niños y adolescentes. Estas narrativas permiten vislumbrar la orientación “preventiva” que los cirujanos estéticos argentinos pretendían imprimir a sus prácticas y el carácter biopolítico de esta empresa. En este marco, sigo la vía trazada por Jarrin (2015) y Schimitt (2021), cuyas publicaciones analizan dos programas públicos brasileros contemporáneos que tuvieron por objetivo facilitar el acceso de niños de escasos recursos a la cirugía de corrección de orejas. Según argumentan, los impulsores de estos programas definen a las otoplastias como intervenciones que atañen a la salud mental y al desarrollo cognitivo de los niños, al evitar la estigmatización que emerge en el contexto de socialización inmediato durante esta etapa de la vida. Asimismo, las autoras muestran la continuidad de estos programas con el discurso eugenésico que permeó el ambiente médico y gubernamental brasilero en la primera mitad del siglo XX, destacando que la normalización de los rostros infantiles se conecta con el perfeccionamiento de la nación.

Según el cirujano plástico argentino Ernesto Malbec (1939a), las otoplastias eran el segundo tipo de intervención más realizada en Argentina, ubicándose por debajo de aquellas que recaían sobre el apéndice nasal. A criterio de este médico, la conformación de las orejas estaba asociada a las características intelectuales y morales de las personas. Esta asociación responde a la relevancia que durante este período asumen la eugenesia y el discurso criminológico de corte biotipológico. Según Stepan (1991), debido a las fuertes conexiones entre Argentina y la Italia de Mussolini en la década de 1930, se observa una expansión de la biotipología entre criminólogos, médicos y profesionales de la salud. Las distintas vertientes de esta teoría proponían sistemas de clasificación de los individuos según su constitución, permitiendo la identificación del “otro” poco apto y peligroso (Armus, 2016). Malbec (1939a, p.1507) muestra una afinidad con esta postura en el siguiente pasaje: “Si bien es cierto que el volumen de la inteligencia no se puede medir por la dimensión de los pabellones, no es menos cierto que la dimensión de los pabellones… es un índice que excepcionalmente fracasa”.

En una posición crítica respecto a las teorías biotipológicas, podemos ubicar al cirujano plástico argentino Jorge Games (1944), que se distancia de los médicos alienistas y criminalistas que conciben las anomalías de los pabellones como caracteres exteriores de una degradación intelectual o moral. En línea con ello, señala que las estadísticas que sustentan esta postura recogen una visión sesgada al registrar solamente la frecuencia con que se producen estos defectos en asilos de alienados y cárceles. La distancia respecto a la perspectiva biotipológica es acompañada por una adopción plena del enfoque psicosomático desarrollado por Zeno y Pizarro Crespo. En este sentido, Games (1944, p.30) señala que “los niños con anomalías morfológicas son víctimas de apodos, puestos por sus compañeros en la época en que comienzan a formar su personalidad, y reaccionan aislándose de ellos y de sus juegos, tornándose huraños, apáticos y retraídos o, no resistiendo la humillación, se hacen agresivos e irascibles”.

En este marco, un consenso básico entre los cirujanos estéticos argentinos de la primera mitad del siglo XX es la necesidad de efectuar las correcciones auriculares antes de que el niño ingrese al ciclo escolar. A criterio de los profesionales, esto obedece a dos motivos. Por un lado, a que el ingreso a la escuela es el momento en que el niño sale de la cápsula protectora familiar y aprende acerca de su defecto a través de insultos, burlas, ostracismo y peleas. Por el otro, a que el medio social hostil actúa como impedimento para el desarrollo conductual y cognitivo de los niños. Malbec (1938) ilustra este punto a través de una narración en la que despliega la carrera moral (Goffman, 2015) arquetípica de un “defectuoso” de las orejas. En ella, el cirujano comienza por señalar que el hogar es un espacio relativamente protegido para el niño y que es cuando este último sale a la calle que padece la hostilidad que “lo indispone contra el medio y genera en él un sentimiento de inferioridad” (Malbec, 1938, p.15).

Una vez ingresado a la escuela, el conflicto se agudiza. Se ve constantemente “perseguido y desplazado” y termina tornándose “huraño, desconfiado, retraído, cuando no se rebela y adopta una actitud inversa, haciéndose entonces violento, agresivo y sanguinario” (Malbec, 1938, p.15). Si continúa asistiendo a la escuela, recibe una “instrucción deficiente”, porque está más atento “a las burlas de sus compañeros que a las indicaciones del maestro” (p.16). Es entonces que estos “niños, futuros hombres del mañana, son retirados prematuramente de los colegios por sus padres” (p.16). La conclusión que Malbec deriva de esta narración arquetípica, es que la temprana corrección estéticas de las orejas puede “evitar toda esta serie de graves inconvenientes que perturban el normal desarrollo de la vida de un niño… y restituir a la sociedad a un miembro que se halla al margen o fuera de ella” (p.18). Según podemos constatar en este pasaje, la normalización de los pabellones auriculares no sólo resultaría clave para prevenir la articulación de comportamientos desviados, sino también como una intervención que contribuiría a reacondicionar a los niños en tanto recursos biopolíticos de la nación (Stepan, 1991).

En el libro *Cirugía plástica*, Lelio Zeno y Emilio Pizarro Crespo (1943) presentan un caso de cirugía estética de orejas en un niño que despliega técnicas de obtención del historial clínico y marcos interpretativos más sofisticados. El caso en cuestión corresponde a un niño de 9 años, escolar de segundo grado, de la ciudad de Rosario que fue operado a fines de la década de 1930. Según dejan sentado en la historia clínica, los padres del niño afirman que el “defecto” de las orejas se hizo visible desde los primeros años, pero que fue recién a los 5 años que el paciente empezó a tomar conciencia del mismo “a causa de las alusiones y pullas de otros chicos que le decían: ‘Orejudo’, ‘Pantalludo’, ‘Orejas de burro’” (Zeno, Pizarro Crespo, 1943, p.305).

Según consignan los autores, el psiquismo del niño se encontraba profundamente afectado: “Conscientemente acusaba fallas graves, a su edad, del carácter (taciturnidad, resentimiento) y de la adaptación (inconducta escolar, incapacidad mental asimilativa etc.)” (Zeno, Pizarro Crespo, 1943, p.307). A nivel inconsciente “sus sueños revelaban ‘agresividad’, impetuosa y reconcentrada” (p.307). En este punto, la influencia del psicoanálisis en la anamnesis se hace patente, a través de una estructura de presentación del caso que intercala una descripción del sueño con su correspondiente interpretación colocada entre paréntesis (Kripper, 2012). A partir de estos síntomas, los médicos confirman psicológicamente la necesidad de la intervención correctora, dado que “las perturbaciones del carácter y de la adaptación social que actualmente presentaba, exigían ser rectificadas con urgencia y como prevención contra reacciones antisociales ulteriores” (Zeno, Pizarro Crespo, 1943, p.307).

A criterio de los autores, los resultados posoperatorios revelaron modificaciones notables en el carácter (la “taciturnidad” cedió paso a una “comunicatividad franca y hasta expansiva”), en la convivencia social (“la agresividad y la rencorosidad” fueron reemplazadas por una “sociabilidad cordial y semieufórica”) y la adaptación: “Visto a los 6 meses de la operación y al finalizar el año escolar, en diciembre de 1939, manifiesta haber pasado con notas sobresalientes… Nos confesó que antes, mientras pretendía estudiar la lección, sólo pensaba en tramar peleas con compañeros que le habían ofendido” (Zeno, Pizarro Crespo, 1943, p.308). A criterio de Zeno y Pizarro Crespo, la cirugía estética de este niño asumió una enorme trascendencia social, al “prevenir probables delitos, agresiones y hasta fracasos profesionales y de adaptación social o amorosa que la persistencia de esos sentimientos hubiera generado casi seguramente cuando mayor” (p.305-308).

Según pudimos constatar en este apartado, la corrección temprana de las orejas de los niños asumiría un papel profiláctico al evitar que los “sentimientos” se consoliden en un “complejo de inferioridad”. Este tipo de cirugías también supondrían un impacto positivo inmediato en el ámbito escolar, al permitir una mejor integración en el grupo de pares y contribuir al rendimiento en materia educativa. Finalmente, las otoplastias aparecen ligadas a un proyecto biopolítico en el que la “normalización” fisonómica de los niños contribuye a mejorar la materia prima con que se construiría el futuro de la nación. Sobre este punto, resulta interesante remarcar el interés que manifiestan algunos de estos cirujanos por persuadir a los funcionarios públicos del área acerca de los beneficios de las correcciones estéticas en la niñez. Esto último, se pone de manifiesto en la dedicatoria del libro *Obra benéfica de la cirugía estética* que el cirujano plástico argentino Codazzi Aguirre (1939, p.1) obsequió a la presidenta del Consejo Nacional de Educación y que está disponible en la Biblioteca Nacional del Maestro de Buenos Aires: “Muchos problemas resueltos por la Cirugía Estética interesan a los educandos, de ahí el gentil obsequio a la digna Pta. del H.C.N. de Educación, la señora Rosa Clotilde Sabattini de Barón Biza. Rosario 26 de mayo de 1958”.

## Cirugía estética al alcance de todos

Según hemos visto hasta aquí, la literatura médica analizada escenifica a la cirugía estética como una terapéutica psicológica. En este apartado me propongo mostrar que esta redefinición de prácticas aparentemente banales en intervenciones que tienen un impacto en la salud de los pacientes viene acompañada de una inquietud de los promotores de la especialidad por asegurar que las clases más desfavorecidas accedan a la cirugía estética. De esta manera, los cirujanos estéticos no sólo emergen como la encarnación del “psicólogo con bisturí”, sino también como “filántropos” que contribuyen al bienestar colectivo garantizando la consumación de un “derecho a la belleza” (Edmonds, 2010).

Cabe destacar que esta definición de la estética en términos de derecho no es una característica exclusiva del desarrollo de la cirugía plástica en Argentina, sino que ha sido ampliamente documentada para el caso de Brasil. Sobre este punto, todas las investigaciones coinciden en destacar la figura del cirujano plástico Ivo Pitanguy (1926-2016), que en 1959 logró abrir el primer centro que ofrecía cirugía plástica para pacientes de bajos recursos en el hospital Santa Casa da Misericórdia, situado en Río de Janeiro. La senda trazada por Pitanguy sería continuada por varios de sus discípulos que, jugando con las ambigüedades del límite entre estética y reconstrucción, instrumentan los recursos del sistema público de salud para subsumirlos a sus intereses privados (Jarrin, 2017).

En Argentina, la preocupación por facilitar el acceso a la cirugía estética se sitúa desde el inicio del proceso de institucionalización de la especialidad. Así, en fecha temprana como 1936, Ernesto Malbec (1936, p.32) señala que la inversión pública en infraestructura y recursos humanos redundaría en una democratización de estas prácticas que alcanzaría a “el pobre, el obrero y al empleado”. Casi cincuenta años después, Malbec retoma este artículo publicado en *La Semana Médica* y hace un balance favorable de lo conseguido en materia de difusión. Destaca que “la prédica constante ante las autoridades públicas para que fueran creados servicios de la especialidad en los hospitales”, permitió que no sólo se operaran los que “cuentan con mayores posibilidades económicas”, sino también “aquellos que antes ni se atrevían a soñar con ser favorecidos por esta conquista de la ciencia” (Malbec, 1983, p.117-118).

Por su parte, el cirujano Pedro Quiroga llevó aún más lejos este afán democratizador, al introducir los servicios de cirugía estética en el Hospital A. Elicagaray, situado en la localidad bonaerense de Adolfo González Cháves a principios de la década de 1940. En un artículo titulado “Cirugía estética rural”, Quiroga (1943, p.1454) señala que enfrentó un ambiente poco propicio para el desarrollo de operaciones “a menudo consideradas de lujo o innecesarias”, pero que aun así logró acercar a este pueblo de ocho mil habitantes una práctica que permite “curar complejos por defectos físicos” (Quiroga, 1943, p.1454). A criterio del autor, la iniciativa del médico rural es esencial para hacer llegar al enfermo del campo la cirugía estética. Según argumenta, los enfermos aquejados de un “tumor o una afección X” pueden ser trasladados a un centro adecuado y cuentan con las “arcas municipales o la colecta de buena voluntad” (p.1454) en caso de ser incapaces de costear el viaje. Por el contrario, “nadie comprendería si a una narigona u orejuda… se le ocurriera hacer una colecta para embellecerse, a costa de una operación que muchos condenan” (p.1455).

La utilización de recursos públicos para financiar intervenciones estéticas parece haber despertado algunos detractores en la comunidad médica. Uno de ellos fue el médico clínico cordobés Jorge Orgaz, cuyo artículo citamos al inicio del segundo apartado. Allí, no sólo define a la cirugía estética como una “ortopedia más para el parece que para el ser” (Orgaz, 29 ene. 1940, p.83), sino que esgrime una crítica al financiamiento público de estas prácticas situándolas en un contexto nacional atravesado por necesidades sanitarias insatisfechas: “Las leguas se suceden y suceden sin una Maternidad ni una Gota de Leche… Y en los suburbios de casi todas las ciudades las casas son pocilgas sin letrinas ni baños… Y en todas partes la tuberculosis cruza barriendo piltrafas individuales y colectivas” (p.83). Orgaz remata esta descripción con una frase contundente, que pone a las claras el despilfarro y el escándalo que implica destinar recursos del Estado al servicio de operaciones banales: “Mientras la cirugía estética empina sobre el paisaje amargo su perfil de Narciso de la técnica” (p.83).

Ahora bien, además de apelar a una alianza con el sector público de salud, la literatura examinada deja entrever que muchos cirujanos plásticos habrían mostrado una disposición a regular sus honorarios de acuerdo a la capacidad de pago de los pacientes. Según el médico legista Royo-Villanova (1958), existe el prejuicio extendido de que la cirugía estética es una práctica onerosa, representación que responde en gran medida a la difusión de las cifras exorbitantes que los astros de la pantalla pagan para rectificarse alguna parte del cuerpo. Sin embargo, este imaginario no está en consonancia con la sensibilidad de los cirujanos que “se hacen pagar según la posición crematística del cliente” (Royo-Villanova, 1958, p.30). Por su parte, el cirujano plástico argentino Estanislao Lluesma Uranga deja aún más claro este punto, apelando a la declaración del cirujano norteamericano Bames: “‘Yo, que opero mucho, no cobro grandes sumas por mis operaciones de estética; la razón es que la inmensa mayoría de mis pacientes, tanto mujeres como hombres, pertenecen a las clases trabajadoras’” (Lluesma Uranga, 1958, p.34-35).

En ocasiones, el afán democratizador parecería llegar aún más lejos, al punto de brindar atención gratuita a pacientes insolventes. Así surge de algunas de las narrativas presentes en la literatura médica analizada. En estos relatos, la gratuidad es abiertamente mencionada, dejando entrever la intencionalidad de los cirujanos plásticos de representar ante los lectores el desinterés económico en el ejercicio de la especialidad. Uno de estos relatos lleva por título “Aeropuerto” (Malbec, 1977), en referencia al emplazamiento donde tuvo lugar un encuentro casual de Ernesto Malbec con un ex paciente. Según relató este último, había sido intervenido de la nariz hacía más de treinta años en Avellaneda (Buenos Aires), y, como provenía de una “familia muy pobre”, Malbec lo había operado gratuitamente en su consultorio. En contraste con esta humilde situación de partida, este hombre había experimentado un cambio de vida radical: “Ahora, mi operado insolvente era un poderoso industrial” (Malbec, 1977, p.133). A pesar de la intrincada red de factores que pueden haber conducido a este cambio, las palabras finales del paciente no dejan dudas acerca de la centralidad que la rectificación gratuita de la nariz había tenido sobre su trayectoria social: “Se da cuenta, doctor, ¿cómo es la vida del hombre? Porque si usted no me hubiese operado entonces la nariz … hoy, en vez de ser un industrial de fuste, a lo mejor andaría con un cajón y varios cepillos lustrando botines” (p.133).

## Consideraciones finales

Comencé este articulo planteando el caso de una mujer parisina que en 1928 sufrió la amputación del tercio inferior de la pierna izquierda a raíz de una cirugía plástica. Los debates suscitados en el marco del proceso judicial y sus derivas en el campo del derecho argentino me permitieron explicitar los distintos posicionamientos en torno al carácter terapéutico de la cirugía estética. Con esta controversia como trasfondo, inicié el recorrido de este artículo. En el primer apartado, procuré reconstruir la historia de la institucionalización de la cirugía plástica en Argentina. Según expuse, aunque a fines del siglo XIX y principios del XX se registran algunas publicaciones sobre la materia, sería en el período de entreguerras el momento en el que surgen los pioneros de la especialidad y las escuelas que se estructurarían en torno a ellos. Estos cirujanos serían centrales en la tarea de producir, divulgar y consolidar institucionalmente a la cirugía plástica latinoamericana y Argentina entre las décadas de 1940 y 1950.

Pioneros y divulgadores de una especialidad médica disputada y poco conocida, sus publicaciones constituyeron los rastros a partir de los cuales procuré reconstruir la empresa cultural que los cirujanos plásticos argentinos asumieron durante la primera mitad del siglo XX. Parte de esta empresa consistió en resignificar e inaugurar una nueva esfera de problemas médicos, mostrando que los “defectos” corporales impactan negativamente en la psicología de los pacientes. Relevante para etiquetar médicamente estos padecimientos psicológicos fue la noción de “complejo de inferioridad”. Acuñada por Alfred Adler a principios del siglo XX, esta teoría gozó de popularidad entre los cirujanos plásticos latinoamericanos y argentinos en las décadas de 1930 y 1940.

Una pieza clave en la empresa cultural antes mencionada fueron las narrativas médicas sobre personas operadas, que presentan los padecimientos de los “defectuosos” suscitados a raíz de la mirada hostil de los “normales” y los cambios positivos experimentados por estas personas en el posoperatorio. Las narrativas protagonizadas por niños y adolescentes operados de las orejas ilustran este punto a la perfección. Por un lado, porque muestran los cambios experimentados por estos jóvenes pacientes, cuyas principales manifestaciones son una mejor integración al medio social y un mayor rendimiento escolar. Por el otro, porque permiten vislumbrar a la cirugía estética como una empresa biopolítica, que previene la articulación de comportamientos disruptivos y genera una integración virtuosa que contribuye al desarrollo de la nación.

Al cierre del artículo, pudimos constatar que la redefinición de la cirugía estética como una intervención que impacta en la salud psíquica de los pacientes viene asociada a diversas iniciativas destinadas a democratizar el acceso a estos servicios médicos. Parte de esta empresa “filantrópica” adquirió expresión en la introducción de estas prácticas en el sector público de salud y la difusión de los “beneficios psicológicos” de la cirugía estética en lugares remotos de la Argentina. Asimismo, asumió concreción en el sector privado, en el que algunos cirujanos estéticos procuraron ajustar sus honorarios a la capacidad económica de los pacientes e incluso brindar atención gratuita.

En suma, esta investigación no solo contribuyó a llenar una laguna en los estudios latinoamericanos en torno a la historia de la cirugía estética, sino también a dejar entrever la relevancia que asume una visión de largo plazo para entender las modulaciones que experimentó el sentido de estas prácticas con el paso del tiempo. En este sentido, lejos de situarlas bajo una lógica de consumo que contribuye a la construcción de proyectos individuales, los cirujanos plásticos argentinos de la primera mitad del siglo XX procuraron medicalizar la experiencia de los pacientes e instalar a la cirugía estética como una práctica normalizadora que contribuye a la consolidación del orden social.
